# Dual-energy CT fusion imaging improves ice-ball visualization in bone during spinal cryoablation in porcine cadavers

**DOI:** 10.1186/s41747-026-00711-4

**Published:** 2026-04-22

**Authors:** Djamel Dabli, Wissem Nabi, Pierre Viala, Julien Frandon, Joël Greffier, Florence Longueville, Marylene Bacle, Jean-Paul Beregi, Maxime Pastor

**Affiliations:** 1 IMAGINE UR UM 103, Montpellier University, Department of Medical Imaging, Nîmes University Hospital, Nîmes, France; 2 Faculty of Medicine, Montpellier Nîmes University, RAM-PTNIM, Nîmes, France

**Keywords:** Cadaver, Cryosurgery, Tomography (x-ray computed), Spine, Swine

## Abstract

**Objective:**

We evaluated *ex vivo* feasibility of ice-ball visualization during bone cryoablation using dual-energy computed tomography (DECT).

**Materials and methods:**

This *ex vivo* study included three porcine cadavers on which cryoablation was performed on four vertebrae. Spectral DECT acquisitions were made at 0 and 10 min after ablation. Conventional images, virtual monoenergetic images (VMIs) at 50 keV, electron density (ED), effective atomic number (Z_eff_) and virtual non-calcium (VNCa) images were generated. Two radiologists assessed visibility and delineation of the ice-ball using a 4-point Likert scale. Quantitative analyses were made on HU VMI, VNCa noted HU*, ED, and Z_eff_ in bone and adjacent tissues. Fused ED and VNCa images were assessed for each vertebra. For one pig, the fused ED and VNCa images were compared with post-ablation magnetic resonance imaging (MRI).

**Results:**

For each pig, the ice-ball was visible in soft tissues on all spectral images except on Z_eff_ images. The best contour delineation was observed on ED images for soft tissues, whereas the ice-ball was detectable in bone only on VNCa images, with poorer but acceptable contour delineation. Significant (*p* ≤ 0.039; effect size (ES) ≥ 0.84) changes in HU and ED were observed for the adjacent soft tissue. For bone, significant (*p* ≤ 0.043; ES ≥ 0.073) changes were found of ED and HU* on VNCa. ED/VNCa fusion improved ice-ball delineation in bone, confirmed by comparison with MRI.

**Conclusion:**

The fusion of spectral ED and VNCa images could show potential in improving the visualization of the ice-ball during bone cryoablation.

**Relevance statement:**

Fusion of electron density and virtual non-calcium images enhances the ice-ball visualization in bone during spinal cryoablation.

**Key Points:**

- The lack of visibility of the ice-ball in the bone during cryoablation represents a limitation for the safety of adjacent structures at risk.
- In bone, the ice-ball was detectable only on virtual non-calcium (VNCa) images, but the quality of its contour delineation was poorer.
- Electron density and VNCa fusion images improved ice-ball visualization and delineation in bone, as confirmed by comparison with MRI.

**Graphical Abstract:**

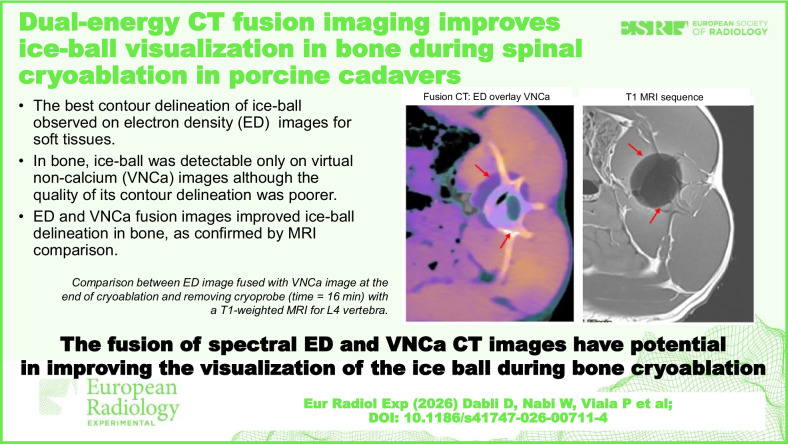

## Background

Bone lesions, whether malignant or benign, represent a significant source of pain and morbidity [1]. Among the malignant lesions, bone metastases affect up to 30% of patients with advanced cancers, particularly in breast, prostate, lung, and myeloma, frequently involving the spine and pelvis [2, 3]. Among the benign lesions, osteoid osteoma is a small, painful, osteoblastic tumor that typically affects young patients [4, 5]; however, other, locally aggressive tumors like giant cell tumors or aneurysmal bone cysts may recur after curettage [6, 7].

Several techniques for managing these tumors are available, including drug treatment, surgery, radiotherapy and interventional radiology. Interventional radiology now plays an increasingly important role, and minimally invasive methods such as percutaneous thermoablation are being quickly developed [8–10]. Bone thermoablation techniques, mainly radiofrequency and cryotherapy, used with a curative or palliative (pain-relieving) intent, offer an effective, safe alternative for inoperable or frail patients, with better tolerability and comparable efficacy to surgery for selected indications [9, 11, 12]. The cryoablation technique is based on the controlled exposure of target tissues to extremely low temperatures, generally below -40 °C. The procedure uses one or more probes (cryoprobes), inserted into the lesion under imaging guidance (computed tomography (CT), magnetic resonance imaging (MRI) or ultrasound), to generate a localized freezing zone known as an “ice-ball”[13, 14]. Most bone cryoablations are performed under CT guidance due to the very limited availability of MRI for interventional procedures [15]. However, on conventional CT, the ice-ball formed within bone tissue is invisible, making it impossible to monitor the progression of the ablation [16, 17]. This can represent a significant challenge when treating lesions that are very close to critical structures such as the spinal cord [11], due to the heightened risk of unintended thermal injury.

Recent studies by Morris et al [18] and Musa J et al [19] suggested the interest of dual-energy CT for visualizing ice-balls in bone using virtual non-calcium images (VNCa). By using two low and high-energy x-ray spectra, the dual-energy CT (DECT) technique can characterize tissues using material decomposition [20]. This decomposition is generally based on two basic materials (*e.g*., water and iodine or calcium). In this way, material-specific images can be generated or one material, such as iodine or calcium (Ca), can be removed from the images to generate virtual non-contrast images or virtual non-calcium images (VNCa). Other types of images can also be generated, such as virtual monoenergetic image (VMI) to enhance contrast at low energy levels and reduce artifacts at high energy, electron density (ED) to measure the number of electrons per unit volume in each voxel and effective atomic number (Z_eff_) images [21]. However, Morris et al [18] and Musa J et al [19] only explored VNCa images with significant blurring, making it difficult to distinguish the ice-ball in bone. As far as we know, no other studies have explored the possibility of visualizing the ice-ball by fusing different spectral images.

The purpose of this *ex vivo* study on porcine cadavers model was therefore to investigate how fusing different spectral images/maps could contribute to ice-ball visualization during bone cryoablation. The hypothesis of this study is based on the potential of spectral images to reveal physical and/or chemical changes in bone tissue subjected to cryoablation treatment.

## Materials and methods

### Animal model

In this single-center *ex vivo* cadaveric experimental study, three porcine cadavers (pig 1, pig 2 and pig 3), weighing 33.8, 33.6 and 33.1 kg, respectively, were used. All pigs were originally obtained as part of a research project aimed at understanding lymphography. All procedures were carried out in accordance with institutional and national guidelines for animal welfare, and with the approval of the ethics committee (see Declarations). All animals were humanely euthanized prior to beginning the study, and the experimental protocol was designed so as not to compromise the integrity of bone structures. The pigs were used for this experiment within 2 h after sacrifice.

### Cryoablation procedure and image acquisitions

The procedure was performed under CT guidance using the Spectral CT7500 (Philips Healthcare, Best, the Netherlands) scanner equipped with dual-layer detector technology for DECT mode. An initial reference non-contrast-enhanced acquisition of the whole spine was made in helical spectral mode prior to trocar placement. The acquisition and reconstruction parameters are provided in Table 1. The trocar was then introduced into each treated vertebra under guidance in interventional CT mode by an interventional radiologist. Once the hole had been made in the vertebra, the trocar was removed and the cryoablation probe introduced. The cryoablation treatment was performed with the ProSense™ (IceCure, Caesarea), Israel) system using liquid nitrogen. All treatments were carried out using the same cryoablation probe (cryoprobe: diameter 3.4 mm, 10 G; length 140 mm).

**Table 1:** CT scan characteristics, acquisition and reconstruction parameters

CT scan	Manufacturer	Philips Healthcare
	Model	Spectral CT7500
	Dual-energy CT technique	Dual-layer detector
	Tube voltage (kV)	120
	Pitch	1.0
	Tube current modulation system	Disabled
Acquisition parameters	Fixed tube current (mA)	362
	Rotation time (s)	0.5
	CTDI_vol_ (mGy) per acquisition	12.00
	Beam collimation (mm)	128 × 0.625
	Mean acquisition time (s)	3.7 ± 0.5
	Reconstruction kernel	YD (sharp)
Reconstruction parameters	Reconstruction algorithm (level)	iDose^4^ (level 4)
	Reconstruction slice thickness (increment) (mm)	3/1.5

Two lumbar vertebrae, L3 and L5, were treated for pig 1, and one vertebra for pig 2 and pig 3, L1 and L4, respectively. For each pig, one spectral CT non-contrast-enhanced acquisition before the cooling cycle at time = 0 min and one at the end of the cooling cycle at time = 10 min were performed with the cryoprobe in place.

Pig 3 was moved to the MRI system to perform a control acquisition at the end of the cryoablation. To prevent the ice-ball from melting during the transport to the MRI system, the cooling cycle was extended to 16 min for this pig. A spectral CT acquisition scan was performed after removing the cryoprobe at 16 min and before moving the pig 3 to the MRI system within 5 min. MRI acquisition was performed on a MAGNETOM Sola 1.5-T system (Siemens Healthineers, Erlangen, Germany) using a T1-weighted turbo spin-echo sequence with the following acquisition and reconstruction parameters: repetition time 600 ms; echo time 7.7 ms; echo train length 3; slice thickness = 4 mm; interslice gap = 0.8 mm (20%); and field of view 359 × 280 mm^2^ (Supplementary Table S1).

The difficulty of accessing the MRI system due to a very full patient examination schedule did not allow for an acquisition to be made for each pig.

### Image processing and visual analysis

For each helical acquisition, conventional images and spectral-based image file were reconstructed. This file was then used to create several spectral images on the AVW server (Philips Healthcare), in particular VMI at 50 keV, ED, Z_eff_ and VNCa. ED images were expressed as the relative ED of each voxel normalized by the ED of water. As for the VNCa imaging, this displays the attenuation of each voxel without the attenuation contribution of calcium-based material. For this image, the newly calculated HU values were expressed as HU*, referring to the HU values of tissues resulting from the material decomposition process leading to the removal of Ca from images. The water/calcium-based material decomposition used was then combined with a suppression index (from 20 to 100%), taking into account the calcium content in the voxel [22]. The calcium suppression index used in this study was 85%, offering the best balance between cortical and spongy bone removal.

Two radiologists viewed all the images at time = 0 min and time = 10 min for all pigs and all treated vertebrae to assess ice-ball visibility in bone and the soft tissue adjacent to the treated vertebrae. The images were assessed by the radiologists according to the quality of ice-ball contour delineation on a 4-point Likert scale: 1 = unacceptable; 2 = acceptable; 3 = good; 4 = excellent.

### Quantitative analyses

**Fig. 1:**
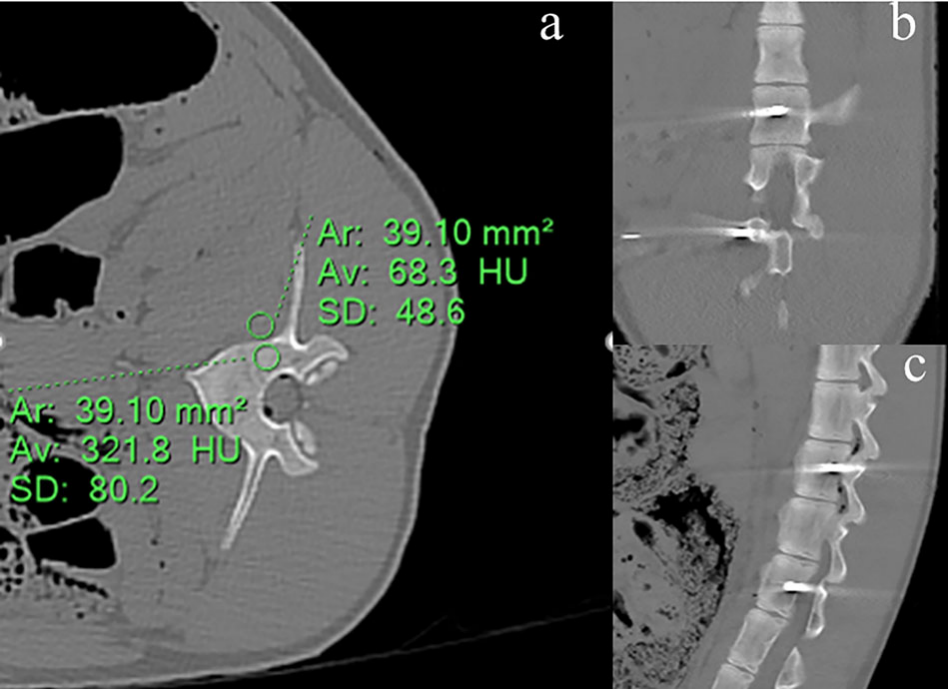
Region of interest placed on treated bone and adjacent soft tissue: **a** axial image, **b** coronal image, **c** sagittal image

For each type of image and all pigs, a 39.1 mm² circular region of interest was placed by one radiologist in the treated area of the vertebra and on the adjacent soft tissue (Fig. 1). Slices distant from those containing the cryoprobe were selected in order to avoid the impact of artifacts caused by the cryoprobe. The values of HU on conventional images, ED on electron density images, Z_eff_ on effective atomic number images and HU* on VNCa images were recorded on 10 consecutive slices on each specific image by placing one region of interest by slice. The values measured on the images acquired at time = 10 min were compared with the initial values measured at time = 0 min. The radiologist was not blind to the time point. The mean differences and associated standard deviation were calculated over all measured data on the three pigs.

### Spectral image fusion and comparison with MRI

To obtain color mapping, ED images were merged with VNCa images for all treated vertebrae. Fusion is a blending of the two images with an adjustable color scale. Fusion of two spectral images, or conventional and spectral images, allows for easy anatomical correlation between different results. These fused images were generated for acquisitions at time = 0 and time =10 min for all pigs and at time = 16 min only for pig 3. In the latter case, the ED/VNCa fusion images at time =16 min were compared with the MRI images.

The ice-ball dimension along the *x*- and *y*-axes was measured on the fused and MRI images by repeating the measurement 10 times in order to assess repeatability. Rigid or deformable registration methods were not used, and the comparison of diameters was based on matched anatomy rather than voxel-level alignment.

### Statistical analysis

Statistical analyses were performed using GMRC Shiny Stats software version 2.0 [23]. The HU, HU*, Z_eff_ and ED data were compared between before and after cryoablation for each image type by using the Wilcoxon signed-rank test for paired samples. A *p*-value ≤ 0.050 was considered significant. In addition, the effect size (ES) values were calculated, that indicates the magnitude of the difference between compared data (small for ES < 0.30, medium for ES from 0.30 to 0.50 and large for ES > 0.50). Regarding the subjective evaluation of the images by the two radiologists, an inter-reader analysis was performed by calculating the κ coefficient for each vertebra. A κ coefficient value greater than 0.6 was considered good agreement, and a value greater than 0.8 was considered excellent agreement.

## Results

### Visual image analysis

**Fig. 2:**
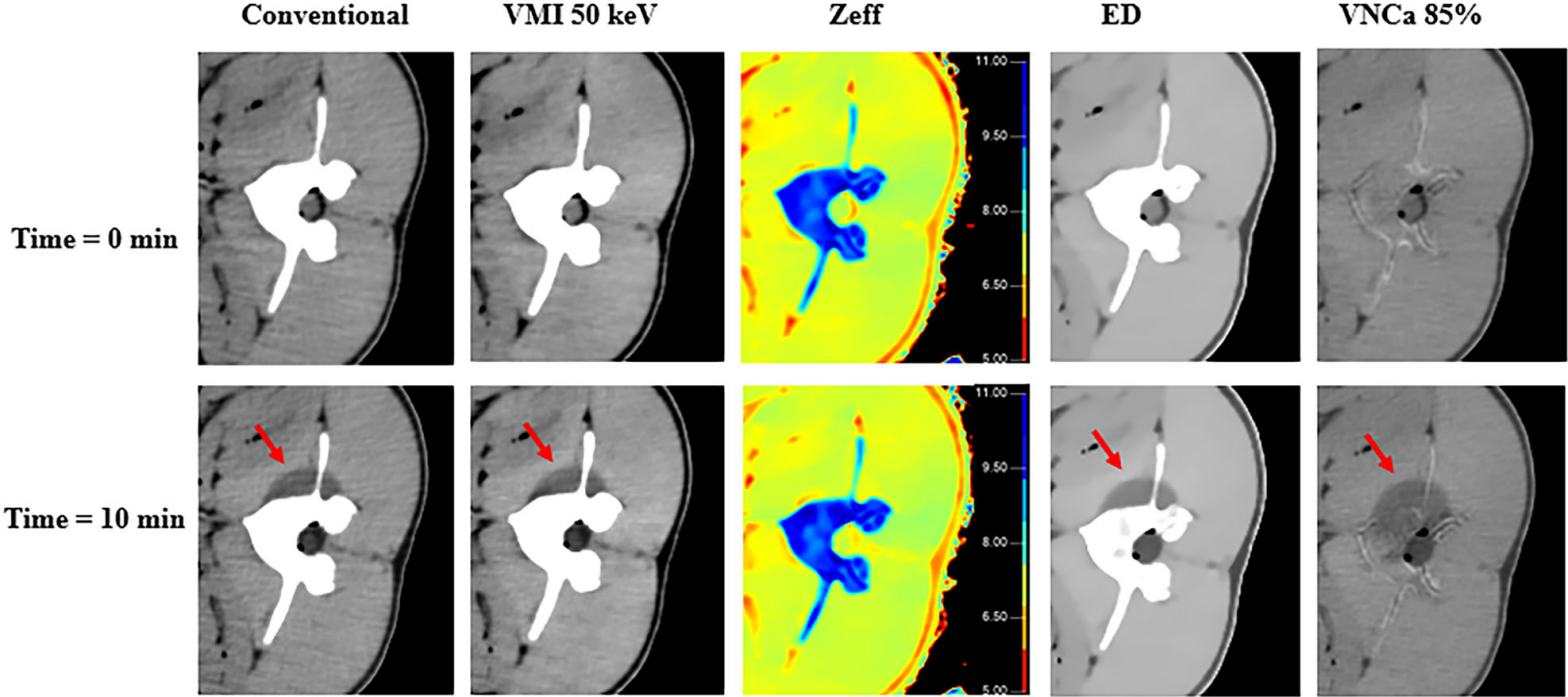
Visual comparison between bone and adjacent soft tissues before (time = 0 min) and at the end of cryoablation (time = 10 min) for L3 vertebra on pig no. 1 and for conventional imaging (window level (WL) 24; window width (WW) 460), virtual monoenergetic imaging (VMI) at 50 keV (WL 49/WW 250), effective atomic number imaging (Z_eff_) (WL 8/WW 6), electron density imaging (ED) (WL 100/WW 25) and virtual non-calcium imaging (VNCa) (WL 32/WW 333). Windowing adapted for soft tissue visualization

Both radiologists confirmed the visibility of the ice-ball at time = 10 min on the soft tissues adjacent to the treated vertebrae on all pigs on all images analyzed, except for the Z_eff_ images. The best ice-ball contour delineation was observed on the ED images ranked as good by both radiologists. Conventional, VMI and VNCa images were ranked as acceptable. Z_eff_’s images were judged unacceptable by both radiologists. The calculated kappa coefficient was equal to 1 for all vertebrae evaluated, indicating excellent agreement between the two radiologists’ readings. Figure 2 shows conventional image, VMIs at 50 keV, Z_eff_, ED and VNCa images obtained at T = 0 min and at 10 min for L3 vertebra on pig no. 1 with windowing optimized for soft tissues.

**Fig. 3:**
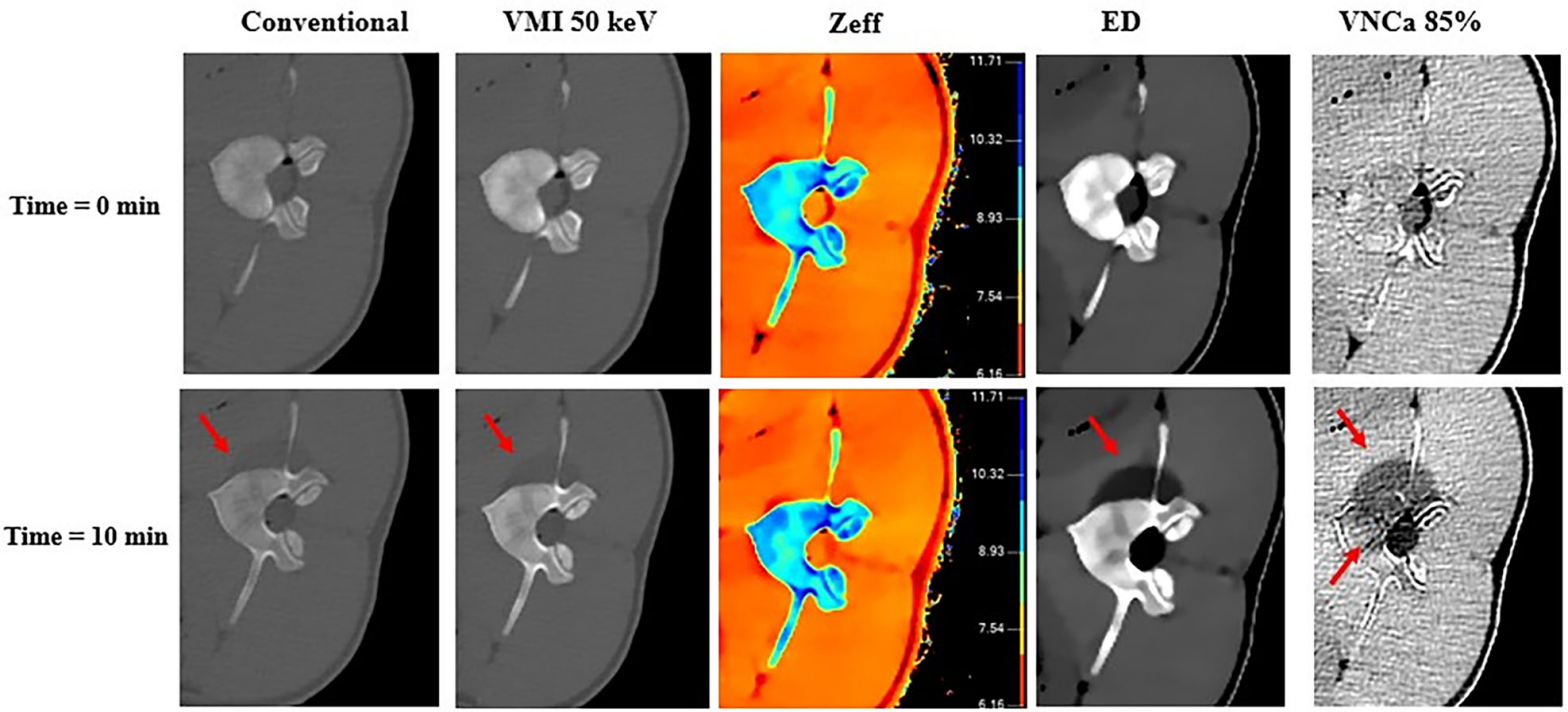
Visual comparison between bone and adjacent soft tissues before (time = 0 min) and at the end of cryoablation (time = 10 min) for L3 vertebra on pig 1 and for conventional imaging (WL 102/WW 1,907), virtual monoenergetic imaging (VMI) at 50 keV (WL 350/WW 2,088), effective atomic number imaging (Z_eff_) (WL 8.9/WW 5.5), electron density imaging (ED) (WL 110/WW 25) and virtual non-calcium imaging (VNCa) (WL 50/WW440).Windowing adapted for bone visualization

For bone tissues, the ice-ball was only visible on the VNCa images, but with a lower quality of ice-ball contour delineation in bone than in soft tissue. The VNCa images were ranked as acceptable by both radiologists, and the other images (conventional, VMI, ED, and Z_eff_) were considered unacceptable. The calculated kappa coefficient was equal to 1 for all vertebrae evaluated. Figure 3 shows the same images with bone tissue windowing.

### Quantitative measurements on conventional and spectral images

Table 2 summarizes the mean quantitative values (HU, Z_eff_, ED, and HU*) measured in soft tissues adjacent to the treated vertebrae across the different conventional and spectral images/maps for each vertebra and animal.

**Table 2:** Results of HU values on conventional images and VMI, HU* on VNCa images, Z_eff_ and relative ED measured before (time = 0 min) and at the end of the cooling cycle (time = 10 min) at the level of ice-ball in soft tissues for each vertebra and each pig

Animal	Vertebra	Cooling time (minutes)	T = 0 s	T = 10 s	Relative difference(%)	*p*-value	Effect size
Pig 1	L3	HU on conventional images	69.6 (66.8; 72.1)	22.3 (18.6; 23.8)	-68.0	**0.011**	0.87
		HU on VMI at 50 keV	65.0 (65.0; 65.3)	29.7 (26; 30.4)	-54.3	**0.005**	0.87
		Effective atomic number (Z_eff_)	7.5 (7.5; 7.6)	7.5 (7.5; 7.5)	0.0	0.098	0.48
		Relative electron density (ED) in %	105 (105; 105.1)	101 (100.6; 101)	-3.8	**0.008**	0.90
		HU* on VNCa with index 85%	46.7 (44.4; 46.7)	3 (-0.1; 4.0)	-93.6	**0.006**	0.90
	L5	HU on conventional images	48.1 (47.7; 50.9)	15.1 (14.7; 15.8)	-68.6	**0.039**	0.84
		HU on VMI at 50 keV	54.3 (54.1; 56.5)	9.6 (9.3; 9.8)	-82.3	**0.005**	0.85
		Effective atomic number (Z_eff_)	7.4 (7.4; 7.4)	7.4 (7.4; 7.4)	0.0	0.779	0.35
		Relative electron density (ED) in %	104.3 (104.3; 104.4)	99.5 (99.4; 99.6)	-4.6	**0.002**	0.91
		HU* on VNCa with index 85%	41.1 (41.0; 41.5)	-8.4 (-9.5; -7.6)	-120.4	**< 0.001**	0.89
Pig 2	L1	HU on conventional images	39 (38.4; 44.6)	6.6 (3.6; 7.2)	-83.1	**0.002**	0.93
		HU on VMI at 50 keV	47.8 (46.4; 48.1)	23.8 (22.8; 24.9)	-50.2	**0.001**	0.89
		Effective atomic number (Z_eff_)	7.4 (7.4; 7.5)	7.2 (7.1; 7.3)	-2.7	0.131	0.30
		Relative electron density (ED) in %	104.2 (104.1; 104.2)	100.4 (100.4; 100.5)	-3.6	**0.036**	0.87
		HU* on VNCa with index 85%	50.1 (45.3; 50.6)	-4.4 (-5.9; -3.7)	-108.8	**0.001**	0.93
Pig 3	L4	HU on conventional images	49.7 (49.1; 51.7)	-2.8 (-3.8; -0.9)	-105.6	**0.001**	0.88
		HU on VMI at 50 keV	69 (64.3; 72.9)	-4.5 (-5.4; -3.9)	-106.5	**0.003**	0.85
		Effective atomic number (Z_eff_)	7.7 (7.6; 7.7)	7.6 (7.6; 7.6)	-1.3	0.067	0.45
		Relative electron density (ED) in %	103.5 (103.5; 103.7)	98.5 (98.5; 98.6)	-4.8	**0.021**	0.86
		HU* on VNCa with index 85%	24 (21.4; 27.0)	-29.6 (-29.8; -28.0)	-223.3	**< 0.001**	0.80

Values are expressed as median (Q1; Q3)

Significant *p*-values are indicated in bold

*ED* Electron density, *VMI* Virtual monoenergetic images, *VNCa* Virtual non-calcium images

No significant differences (*p* ≥ 0.067) were observed between the Z_eff_ measured at time = 0 min or time = 10 min (-1.3 ± 1.0%). For the other data measured, a significant difference was observed in the HU measured on conventional images at time = 0 and time = 10 min (-81.3 ± 17.6%; *p*-values from 0.001 to 0.039). This difference was significant for HU values on VMI (-73.3 ± 26.3%; *p*-values from 0.001 to 0.005), for ED (-4.2 ± 0.6%; *p*-values from 0.002 to 0.036), and for HU* (-136.5 ± 58.9; *p* < 0.001), respectively. The ES values were higher than 0.50 for all compared data except for Zeff.

For the bone tissue in each treated vertebra (Table 3), no significant differences were observed regarding the HU values measured on conventional images (-8.4 ± 4.8%; *p*-values from 0.068 to 0.630) or on VMIs at 50 keV (-0.7 ± 10.0%; *p*-values from 0.088 to 0.2113) or Z_eff_ (-0.0 ± 0.8%; *p*-values from 0.125 to 0.684). The difference was significant for ED (-3.5 ± 1.0%; *p*-values from 0.028 to 0.043) and HU* on VNCa images (-125.1 ± 61.6%; *p*-values from lower than 0.001 to 0.023). The ES values were higher than 0.50 for all compared data except for Zeff.

**Table 3:** Results of HU values on conventional images and VMI, HU* on VNCa images, Z_eff_ and relative ED measured before (time = 0 min) and at the end of the cooling cycle (time = 10 min) at the level of ice-ball in bone for each vertebra and each pig

Animal	Vertebra	Cooling time (minutes)	T = 0 s	T = 10 s	Relative difference (%)	*p*-value	Effect size
Pig 1	L3	HU on conventional images	266.0 (258.0; 268.0)	252.0 (248.0; 255.0)	-5.3	0.125	0.69
		HU on VMI at 50 keV	466.0 (455.0; 470.0)	510.0 (490.0; 514.0)	9.4	0.063	0.84
		Effective atomic number (Z_eff_)	10.0 (9.9; 10.1)	10.1 (10.0; 10.1)	1.0	0.684	0.21
		Relative electron density (ED) in %	115.2 (115.1; 115.3)	112.8 (112.4; 112.9)	-2.1	**0.038**	0.81
		HU* on VNCa with index 85%	36.2 (35.9; 37.5)	-8.3 (-10.7; -4.9)	-122.9	**0.023**	0.80
	L5	HU on conventional images	235.2 (227.2; 237.8)	223.1 (225.15; 225.2)	-5.1	0.630	0.83
		HU on VMI at 50 keV	390.8 (380.1; 394.6)	412.1 (401.4; 415.7)	5.5	0.213	0.86
		Effective atomic number (Z_eff_)	9.8 (9.7; 9.9)	9.8 (9.7; 9.8)	0.0	0.643	0.42
		Relative electron density (ED) in %	116.8 (115.6; 117.1)	111.7 (111.6; 111.7)	-4.4	**0.043**	0.73
		HU* on VNCa with index 85%	36.3 (36.1; 37.0)	7.0 (4.0; 8.2)	-80.7	**0.003**	0.95
Pig 2	L1	HU on conventional images	357.2 (347.5; 573.6)	329.3 (320.8; 342.8)	-7.8	0.125	0.69
		HU on VMI at 50 keV	526.6 (512.3; 543.8)	460.2 (448.8; 462.7)	-12.6	0.138	0.83
		Effective atomic number (Z_eff_)	10.2 (9.9; 10.3)	10.3 (10.2; 10.3)	-1.0	0.625	0.20
		Relative electron density (ED) in %	120.6 (119.7; 122.4)	116.3 (116.2; 117.3)	-3.6	**0.034**	0.81
		HU* on VNCa with index 85%	83.0 (82.8; 83.5)	13.3 (12.9; 17.7)	-84.0	**< 0.001**	0.92
Pig 3	L4	HU on conventional images	407.0 (403.0; 411.8)	344.5 (343.9; 346.8)	-15.4	0.068	0.85
		HU on VMI at 50 keV	481.6 (473.4; 485.2)	458 (457.1; 460.5)	-4.9	0.088	0.89
		Effective atomic number (Z_eff_)	10.5 (10.4; 10.5)	10.5 (10.5; 10.5)	0.0	0.125	0.35
		Relative electron density (ED) in %	120.5 (120.6; 120.9)	115.8 (115.7; 115.9)	-3.9	**0.028**	0.83
		HU* on VNCa with index 85%	29.3 (27.7; 29.9)	-33.1 (-35.9; -31.6)	-213.0	**< 0.001**	0.88

Values are expressed as median (Q1; Q3)

Significant *p*-values are indicated in bold

*ED* Electron density, *VMI* Virtual monoenergetic images, *VNCa* Virtual non-calcium images

### Spectral image fusion and comparison with MRI

**Fig. 4:**
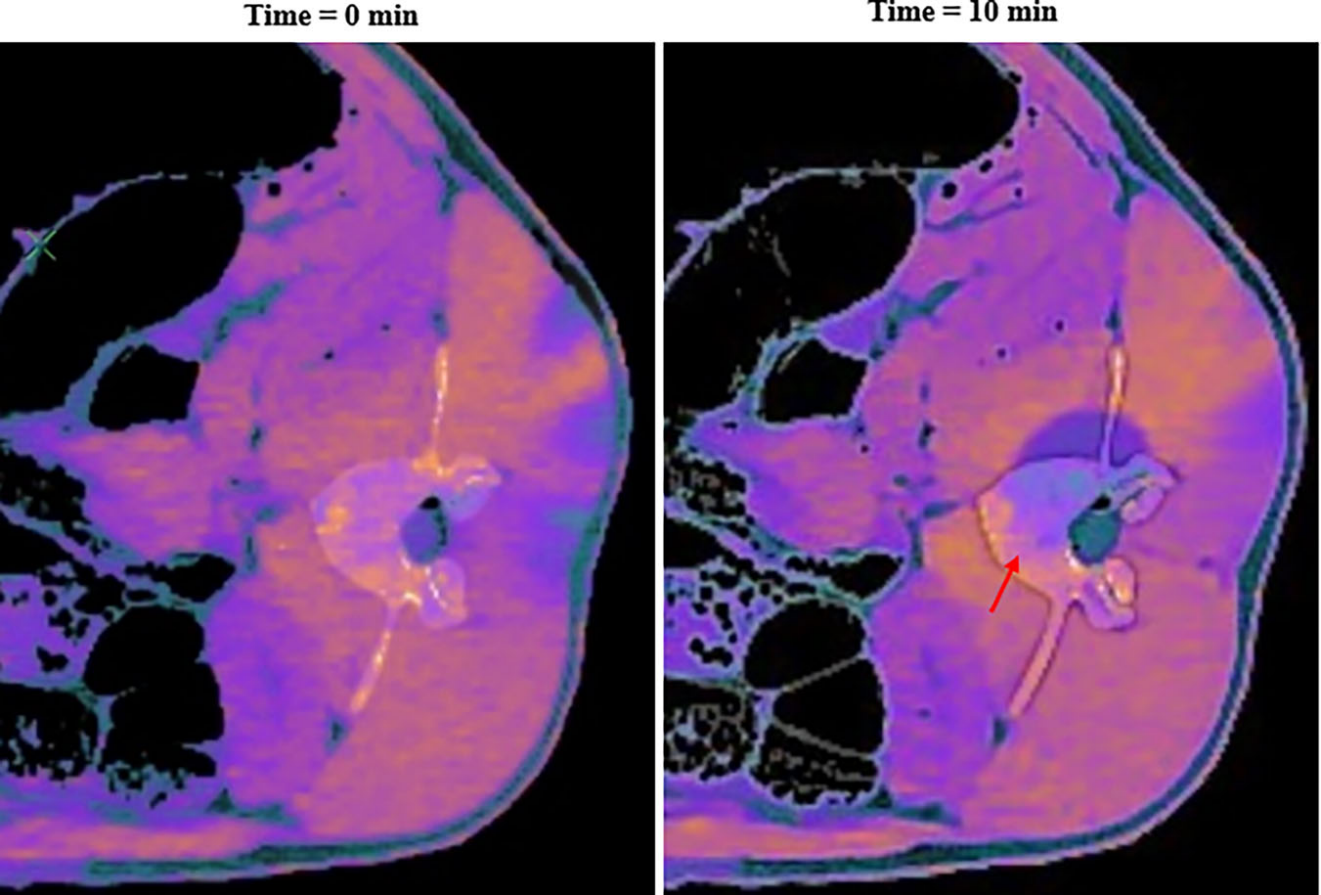
Visual comparison between electron density image fused with virtual non-calcium image before (time = 0 min) and at the end of cryoablation (time = 10 min) for L3 vertebra on pig 1

Figure 4 shows the fusion of ED and VNCa images for the same case as shown in Figs. 2 and 3. Both radiologists rated this image as excellent for visualizing the contours of the ice-ball on soft tissues and as good for bone.

**Fig. 5:**
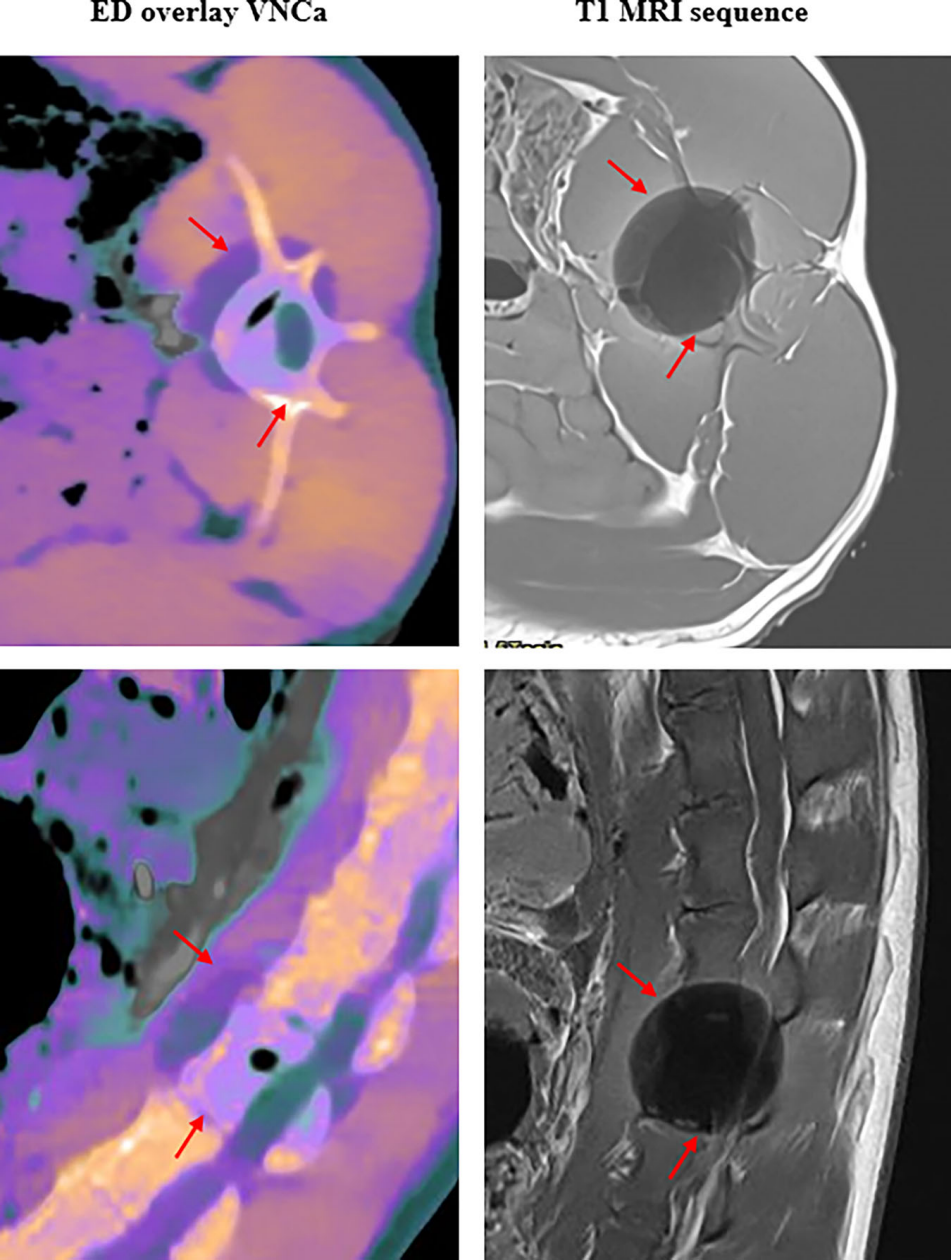
Visual comparison between electron density image fused with virtual non-calcium image at the end of cryoablation and removing cryoprobe (time = 16 min) with a T1-weighted magnetic resonance imaging for L4 vertebra on pig 3: upper side for the axial image and reverse side for the sagittal image

A comparison between the fused ED/VNCa images for the cooling cycle of 16 min of the pig 3 and a T1-weighted MRI sequence is shown in Fig. 5. MRI images confirmed the presence of the ice-ball, whose shape and position were consistent with the image observed on the fused ED and VNCa spectral CT images. The dimensions of the ice-ball measured on the axial section of the merged ED and VNCa images were 29.8 ± 0.7 mm along the *x*-axis and 33.8 ± 0.8 mm along the *y*-axis. These dimensions were 30.2 ± 0.6 mm and 33.2 ± 0.7 mm, respectively, on the MRIs. The repeatability of measurements was 2.3% on ED/VNCa fusion images and 2.0% on MRI images. The differences observed between the X and Y dimensions in the two images were 1.1% and 1.6%, respectively.

## Discussion

The initial results found in this exploratory *ex vivo* study on 3 pigs showed that the ice-ball could be visualized within the vertebral bone by fusing ED with VNCa spectral images. The conspicuity of the ice-ball was rated better than on VNCa images alone. Comparison with MRI images for one vertebra showed that the shape and size of the ice-ball visualized on ED images fused with VNCa were consistent with those obtained on MRIs.

Visual analysis of spectral images performed by radiologists showed that the ice-ball was clearly visible in the soft tissue adjacent to the treated vertebrae in all spectral images/maps generated except for the Z_eff_ images. These results were corroborated by the quantitative measurements made on the images, where no significant variations (ES < 0.50) in Z_eff_ were found for each pig. This might be explained by the fact that freezing the soft tissue does not alter its chemical composition. However, the HU values of the soft tissue measured on conventional images and on the VMI at 50 keV before and after cryoablation decreased significantly with ES values > 0.50. Similar results were observed on the ED images and on the VNCa, resulting in a hypodense signal on the images. The decrease in attenuation of the soft tissues containing ice-balls could be explained by the change in the state of matter. In ice-balls, the molecules of water are arranged as a network of crystals with a higher intermolecular space than in normal soft tissues. As there are more empty spaces—and therefore fewer atoms per unit volume—this would explain the decrease in electron density in ED images. Since x-ray attenuation is proportional to electron density, the decrease in electron density also results in a decrease in HU values. This decrease in HU as the soft tissues decrease in temperature has been reported in a previous study on conventional images [24]. Nevertheless, compared to this previous study, the present study provides additional insight by incorporating ED images into the analysis. The results demonstrate that radiologists have observed that ice-ball conspicuity was better on ED images than on conventional images and VMIs at 50 keV.

Regarding vertebral bone, visual analysis showed no apparent ice-balls on all conventional and spectral images apart from the VNCa images. This was confirmed by quantitative measurements where no significant variations were found for conventional, VMI at 50 keV and Z_eff_ images between before and after cryoablation but significant variations for the HU* values measured on VNCa images. However, the high ES values (higher than 0.50) for VMI at 50 keV results suggests a substantial difference, indicating that the lack of significance could be related to the low number of measurement points limited by the number of usable slices due to probe artifacts. Although the variations in relative electron density values in the ice-ball on the ED images were weak, the differences between before and after cryoablation were significant with a high ES value (higher than 0.50). In fact, electron density in bone is naturally high at normal temperatures, reflecting a very small intermolecular gap. The statistically not significant variation in HU in bone for conventional images can be explained by the fact that bone is highly dense due to its calcium content. Thus, a decrease in temperature does not significantly increase this gap. However, vertebrae are also vascularized, hydrated tissues, and therefore contain other elements of low electron density. These elements undergo changes similar to those observed in soft tissues under the effect of cooling by cryoablation. Consequently, removing the calcium contribution on the vertebral bone using VNCa images made it possible to note these changes visually and quantitatively. However, the visualization quality of the ice-ball in bone was considered poorer than in soft tissue, particularly in terms of conspicuity. These results are consistent with the studies by Morris et al [18] and Musa J et al [19] despite the fact that a different DECT platform was used by Morris et al [18]. In this study, we defined a Ca suppression index of 85% because it presented the best compromise between removing Ca in a sufficient number of voxels while preserving the anatomy of the vertebrae, thus maintaining structural landmarks.

Based on visual and quantitative analysis of the various conventional and spectral images, it was decided to fuse the ED images with the VNCa images in order to enhance the changes in soft tissue and bone with a specific color mapping. The results confirmed the visibility of the ice-ball, including in the bone, and, most importantly, a clear improvement in its conspicuity. The MRI images acquired on pig 3 provided a reference image to compare the size and location of the ice-ball with the fused ED and VNCa color image. The MRI images clearly showed the presence of the ice-ball in the vertebral bone and adjacent soft tissues. The ice-ball appeared as a hypointense signal [25] due to the absence of proton mobility caused by the cooling effect. Finally, the shape and position of the ice-ball obtained with 16 min cooling duration after removing the cryoprobe are consistent between fused ED and VNCa color map and the MRIs. The differences observed between the *x* and *y* dimensions in the two images were within the expected measurement error (2%).

In bone cryoablation, particularly in spinal and pelvic locations, it is essential to accurately visualize the ice-ball to ensure procedural safety, given the close proximity of critical structures such as the spinal cord, nerve roots, neurovascular plexuses, and adjacent viscera [26, 27]. Although MRI is more sensitive at detecting ice-ball formation, it remains poorly accessible intra-procedurally due to its logistical and technical constraints, requiring hybrid operating environments, specialized coils, and longer acquisition times. As a result, many bone cryoablations are performed under CT guidance without direct visualization of the freezing front within bone, potentially increasing the risk of unintentional extension toward sensitive structures such as the spinal cord or exiting nerve roots. In this context, the fusion of VNCa and ED spectral CT images, as proposed in this study, may overcome this critical limitation. Beyond the interest of monitoring the procedure, these results would open up the possibility to characterize the growth of ice-balls in bone and thus develop predictive models to simulate cryoablation treatment and plan it beforehand to optimize treatment, as has already been developed for other anatomical regions [28].

The main challenge to overcome in better visualizing the ice-ball during procedures is the reduction of metallic artifacts caused by the cryoprobe. As stated by Borgbjerg et al [29], dual-energy CT can help reduce probe metallic artifact in soft tissue locations. However, the study by Morris et al [18] reported that in bone location, VNCa images remained significantly blurred and compromised by these artifacts, even when metal artifact-reduction algorithms are used. These artifacts were also present in the images obtained in this study, making it more challenging to visualize the ice-ball during the cooling cycle (small ice-ball size). To fully optimize visibility, it is advisable to integrate artifact-reduction techniques such as tilt the CT scan to align it with the axis of the cryoprobe, use highest energy levels on VMIs and use a metal artifact-reduction algorithm adapted to cryoablation probe material, as most current algorithms are optimized for orthopedic devices [30, 31]. A recent study has shown that photon-counting scanners improve metal artifact removal [32]. Finally, the second challenge is to optimize the workflow and reconstruction time of spectral images in order to make them compatible with the need for real-time monitoring during cryoablation [33].

This study has certain limitations. It was carried out using a single scanner model on a small sample size with the parameters used in our center, and the results could be different on other CT systems with other acquisition and reconstruction parameters and other spectral performance. However, this is an initial exploratory study aimed at assessing the feasibility of using spectral images to improve the visualization of the ice-ball in bone. The assessment of the quality of the ice-ball edges was performed subjectively by two radiologists using a subjective 4-point scale and not by quantitative and objective metrics. The impact of cryoprobe artifacts on spectral images has not been studied in detail. Nevertheless, the purpose of this study is to prove the concept of visualizing ice-ball on spectral images. Optimizing the impact of artifacts should be the subject of a more specific study. The animal model used is close to human but does not allow us to state that the results will be identical in humans. Specifically, water density in animals may differ from that in humans due to post-mortem conditions and a delay of 2 h in CT/MRI acquisition after death. MRI acquisition was carried out in only one pig because of practical difficulties in accessing the MRI. However, the aim of this comparison was to confirm the presence of the ice-ball at the end of the procedure, given that its visibility has already been validated in the literature.

In conclusion, the fusion of ED and VNCa spectral images obtained on DECT enhances the visualization of the ice-ball during bone cryoablation, in particular in the bone compartment. The position and dimensions of the ice-ball on ED/VNCa fusion color images were in agreement with measurements performed on MRI.

## Supplementary information

**Additional file 1: Table S1:** MRI system characteristics, acquisition and reconstruction parameters.

## Data Availability

The datasets analyzed during the current study are available from the corresponding author upon reasonable request.

## Abbreviations

CT: Computed tomography
DECT: Dual-energy computed tomography
ED: Electron density
MRI: Magnetic resonance imaging
VMI: Virtual monoenergetic imaging
VNCa: Virtual non-calcium
Z_eff_: Effective atomic number
