# Supplementary material for: Dual-energy CT fusion imaging improves ice-ball visualization in bone during spinal cryoablation in porcine cadavers
Source: Eur Radiol Exp. 2026 Apr 22;10:51. doi: 10.1186/s41747-026-00711-4 (PMC13103238; doi:10.1186/s41747-026-00711-4)
Supplement: Supplementary file 1 — Additional file 1: Table S1: MRI system characteristics, acquisition and reconstruction parameters. [file 41747_2026_711_MOESM1_ESM.docx]

**Supplementary material**

**Table SM 1 :** MRI system characteristics, acquisition and reconstruction parameters

| MRI system | Manufacturer | Siemens Healthineers |
| --- | --- | --- |
|  | Model | MAGNETOM Sola 1.5 Tesla |
| Acquisition parameters | Sequence | T1-weighted Turbo Spin Echo |
|  | Repetition time (ms) | 600 |
|  | time to echo (ms) | 7.7 |
|  | echo train length | 3 |
|  | slice thickness (mm) | 4 |
| Reconstruction parameters | interslice gap (mm) | 0.8 mm (20%) |
|  | field of view (mm x mm) | 359 x 280 |
